# Maternal mental illness and child atopy: a UK population-based, primary care cohort study

**DOI:** 10.3399/BJGP.2022.0584

**Published:** 2023-10-03

**Authors:** Cemre Su Osam, Holly Hope, Darren M Ashcroft, Kathryn M Abel, Matthias Pierce

**Affiliations:** Centre for Women’s Mental Health, Faculty of Biology, Medicine and Health, University of Manchester, Manchester.; Centre for Women’s Mental Health, Faculty of Biology, Medicine and Health, University of Manchester, Manchester.; Centre for Pharmacoepidemiology and Drug Safety, Faculty of Biology, Medicine and Health, and National Institute for Health and Care Research Greater Manchester Patient Safety Translational Research Centre, University of Manchester, Manchester.; Centre for Women’s Mental Health, Faculty of Biology, Medicine and Health, University of Manchester; Greater Manchester Mental Health NHS Foundation Trust, Manchester.; Centre for Women’s Mental Health, Faculty of Biology, Medicine and Health, University of Manchester, Manchester.

**Keywords:** allergy and immunology, asthma, eczema, mental disorders, primary health care

## Abstract

**Background:**

The number of children exposed to maternal mental illness is rapidly increasing and little is known about the effects of maternal mental illness on childhood atopy.

**Aim:**

To investigate the association between maternal mental illness and risk of atopy among offspring.

**Design and setting:**

Retrospective cohort study using a UK primary care database (674 general practices).

**Method:**

In total, 590 778 children (born 1 January 1993 to 30 November 2017) were followed until their 18th birthday, with 359 611 linked to their hospital records. Time-varying exposure was captured for common (depression and anxiety), serious (psychosis), addiction (alcohol and substance misuse), and other (eating and personality disorder) maternal mental illness from 6 months before pregnancy. Using Cox regression models, incidence rates of atopy were calculated and compared for the exposed and unexposed children in primary (asthma, eczema, allergic rhinitis, and food allergies) and secondary (asthma and food allergies) care, adjusted for maternal (age, atopy history, smoking, and antibiotic use), child (sex, ethnicity, and birth year/season), and area covariates (deprivation and region).

**Results:**

Children exposed to common maternal mental illness were at highest risk of developing asthma (adjusted hazard ratio [aHR] 1.17, 95% confidence interval [CI] = 1.15 to 1.20) and allergic rhinitis (aHR 1.17, 95% CI = 1.13 to 1.21), as well as a hospital admission for asthma (aHR 1.29, 95% CI = 1.20 to 1.38). Children exposed to addiction disorders were 9% less likely to develop eczema (aHR 0.91, 95% CI = 0.85 to 0.97) and 35% less likely to develop food allergies (aHR 0.65, 95% CI = 0.45 to 0.93).

**Conclusion:**

The finding that risk of atopy varies by type of maternal mental illness prompts important aetiological questions. The link between common mental illness and childhood atopy requires GPs and policymakers to act and support vulnerable women to access preventive (for example, smoking cessation) services earlier.

## INTRODUCTION

Mothers play a key role in the development of offspring: from pregnancy to early adulthood, mothers are likely to be the primary caregivers.

Accumulating evidence suggests that children exposed to maternal mental illness have poorer physical health than unexposed children.^1^ Some evidence highlights the risk of atopic diseases, such as asthma, eczema, allergic rhinitis, and food allergies,^2^^–^^4^ which are increasingly prevalent, now occupying some of the commonest global health problems among children.^5^ For example, asthma affects almost 14% of children worldwide and, in the UK, one in 11 children receive asthma treatment, costing the NHS approximately £1 billion per year.^6^ The prevalence of eczema, allergic rhinitis, and food allergies is also increasing:^7^ 20% of the UK population has ≥1 atopic disease.^8^ Currently, almost 25% of children in the UK live with a mother experiencing mental illness,^9^ which the authors’ research group estimated was associated with an excess annual NHS cost of £560 million in health utilisation.^10^

Atopic disease in children is common and associated with common mental illness (CMI) among young people,^11^ and maternal mental illness is linked to increased risk of offspring atopy.^1^ This points to common aetiological mechanisms shared between depression, anxiety, and atopic disease.^12^ Evidence for such mechanisms remains limited by relatively small samples, use of self-report measures of mental illness, and exposures/outcomes restricted to maternal CMI and childhood asthma.^13^^–^^20^ Further, studies fail to account for potential confounders such as younger maternal age, or poverty and other socioeconomic factors; or examine environmental pathways potentially leading to atopy risk, including poor antenatal care, maternal smoking, (both while pregnant and as a parent), fetal growth restriction, or maternal antibiotic exposure. Finally, if environmental factors are important in the aetiology, in offspring with atopy whose mothers have serious mental illness (SMI) and higher levels of adversity they should experience higher risk of atopic disease compared with their peers whose mothers are well or have CMI only.^21^

This study used a large representative population-based cohort of mothers and children to compare the risk of atopic diseases in children of mothers with mental illness and mothers without mental illness. It was hypothesised that:

- children of mothers with mental illness would have a higher risk of developing atopic diseases compared with children of mothers without mental illness;
- the risk of atopic diseases would be higher among children exposed to serious maternal mental illness (affective and non-affective psychosis) compared with more common maternal mental illnesses (depression and anxiety); and
- the risk of admission to hospital for asthma or food allergy (indicating a serious atopy event) would be higher among children of mothers with mental illness than mothers without mental illness.

**Table:** How this fits in

Prior reports of increased risk of asthma and allergic rhinitis in children of mothers with depression or anxiety has excluded important confounders and not considered potential environmental pathways. This study found an increased risk of asthma and allergic rhinitis among children exposed to maternal depression or anxiety, and explores the role that maternal smoking plays (both smoking while pregnant and/or as a parent) in childhood atopy. Future research, to test if the specific association seen here means maternal depression/anxiety causes childhood atopy, is required. Tailoring communication and preventive services to the health needs of women experiencing mental illness would benefit both women and their children.

## METHOD

### Data source

This retrospective cohort analysis utilised the Clinical Practice Research Datalink (CPRD) GOLD with anonymised primary healthcare records for about 10% of UK general practices.^22^ Clinical event data are collected routinely on consultations (including diagnosis), referrals, and prescriptions.

The analysis cohort was constructed using the CPRD mother–baby link, an algorithmic linkage of children and mothers based on pregnancy, delivery, and birth records, and a household identifier.^23^

The hospital episode statistics (HES) dataset holds anonymised electronic healthcare records of all NHS (free at the point of access) hospital visits in England; approximately 75% of English practices registered with CPRD GOLD consented for their patients’ records to be linked.^22^

Socioeconomic data are based on Index of Multiple Deprivation (IMD) linked to GP practice postcode. The IMD is a rank score of area-level deprivation, divided into quintiles, derived using seven domains including: income, employment, education, health and disability, crime, barriers to housing and services, and the lived environment.

### Cohort selection

**Figure 1.**
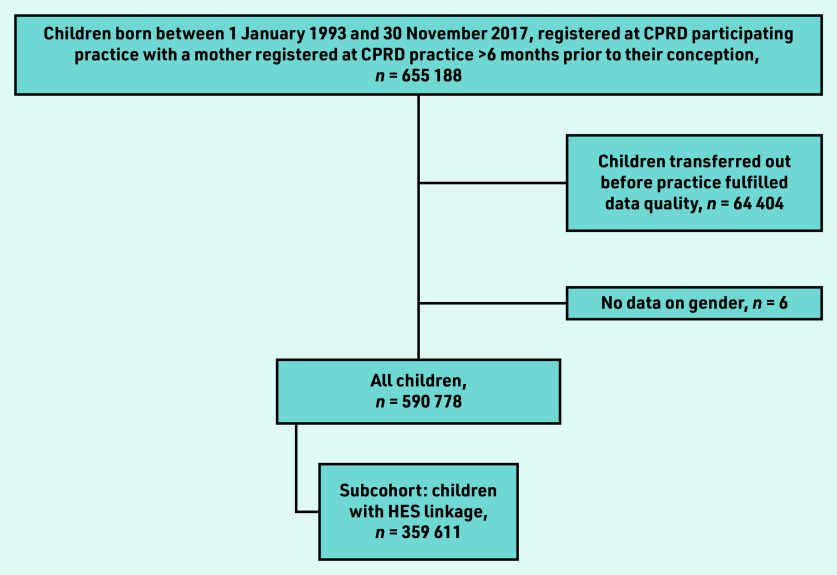
*Cohort selection process. CPRD = Clinical Practice Research Datalink. HES = hospital episode statistics.*

Eligible children included those born between 1 January 1993 and 30 November 2017, whose mother was registered at a general practice for >6 months before the child’s date of birth (Figure 1).

Follow up started on the latest date of the: child’s birth; the practice started collecting data deemed ‘up-to-standard’; child’s registration; study start date (1 January 1993). Follow up ended at the earliest date of the: child or mother’s death or transfer out of general practice; child’s 18th birthday; end of data collection; and study end date (31 December 2017). This included 590 778 children and 428 924 mothers with an average follow up of 5.24 person–years. To investigate the risk of secondary healthcare utilisation for atopic diseases among children exposed to maternal mental illness, 359 611 of this cohort were selected who had HES linkage.

### Exposure

Children’s exposure to maternal mental illness was identified using primary care data on each mother’s recorded diagnoses, symptoms, prescriptions, and referral to external services (see Abel *et al*^9^ for further details). Children were defined as exposed from the first record of maternal depression, anxiety, non-affective and affective psychosis, alcohol or substance misuse, eating disorder, or personality disorders from 6 months before pregnancy until the end of follow up.

For this analysis, mental illnesses were grouped into: CMI (anxiety and depression), SMI (non-affective and affective psychosis), addiction (alcohol and substance misuse), and ‘other’ (eating and personality disorders).

### Outcome

Atopic diseases included incident diagnosis of childhood asthma, eczema, allergic rhinitis, or food allergy. Clinical codes identifying diagnoses are available on github.^24^

To analyse the risk of admission to hospital for serious atopic disease all records were captured of hospital admission (day, night, planned, or unplanned) in HES where there was an International Classification of Diseases diagnosis of either asthma or food allergy.^25^

### Covariates

Data on maternal age at birth, child sex, maternal history of atopic disease, antibiotic use during pregnancy, and smoking status were extracted from the CPRD. Validated HES data on child ethnicity (Asian/British Asian, Black/Black British, White, Mixed, and Other) were used and supplemented with CPRD data if missing.^26^ CPRD data on the practice’s region (North East, North West, Yorkshire & The Humber, East Midlands, West Midlands, East of England, South West, South Central, London, South East Coast, Northern Ireland, Scotland, and Wales) and IMD were used.

### Statistical analysis

The risk of atopic disease (time to first event) for children exposed and unexposed to maternal mental illness was calculated and presented as incidence rates (events per 1000 person–years). Cox proportional hazard models were used to compare risks for maternal mental illness in the exposed and unexposed children, presented as hazard ratios. Potential confounders were adjusted for: maternal atopy history, antibiotic use during pregnancy, and age at birth; and child sex, ethnicity, delivery year, deprivation quintile, and region in model 1. The authors considered smoking on the causal pathway; therefore, a second model also included it as a covariate (model 2).

In the cohort with HES linkage, the time to an atopy hospital admission (asthma and food allergy) was compared in children in those exposed to mothers with and without maternal mental illness.

In all regression analyses, continuous variables were centred and a squared term included. Clustering by maternal sibships was accounted for by calculating the standard error using the Huber–White sandwich estimator.^27^ Data were analysed using Stata (SE 15.0).

This study meets the requirements of the RECORD statement.^28^ In this study the authors considered hazard ratios (HRs) where the 95% confidence interval (CI) did not cross 1.0 and a significance *P*-value <0.05 to be more conclusive or stronger evidence of an association. HRs were reported to two decimal places.

### Sensitivity analysis

Although it is understood that some cases of infantile eczema can be severe and if untreated may require secondary care, the authors were concerned that any association between maternal mental illness and childhood eczema might be explained by mild, highly prevalent and time-limited infantile eczema. A sensitivity analysis was therefore conducted that started follow up from 3 years of age.

## RESULTS

The analysis included 590 778 mother–child pairs (Figure 1) and a total of 3 840 135 person–years follow up (median time in analysis: 5.24 years; interquartile range 2.12–10.01) with a mean maternal age of 30.3 years (standard deviation 5.8) and 48.7% (*n* = 287 682/590 778) female offspring. Over follow up, 38.6% (*n* = 227 832/590 778) of children were exposed to maternal CMI and 35.9% (*n* = 212 295/590 778) maternal smoking (Table 1).

**Table 1.** Characteristics of mother–child cohort, children born between 1 January 1993 and 31 December 2015 (*N* = 590 778)

**Characteristic**	***n***	**%**
**Child sex**		
Female	287 682	48.7
Male	303 096	51.3

**Child ethnicity**		
Asian/British Asian	18 980	3.2
Black/Black British	8891	1.5
Mixed	11 656	2.0
Other	5334	0.9
White	363 226	61.5
Unknown	182 691	30.9

**Child atopic disease**		
Any	217 568	36.8
Asthma	49 893	8.4
Eczema	180 933	30.6
Allergic rhinitis	33 097	5.6
Food allergies	9753	1.7

**Maternal age at delivery, years**		
<20	21 488	3.6
20–29	232 765	39.4
30–39	308 106	52.2
>40	28 419	4.8

**Maternal mental illness**		
CMI	227 832	38.6
SMI	4432	0.8
Addiction	7745	1.3
Other^a^	6188	1.0

**Maternal smoking (during child’s life)**		
Never	292 312	49.5
Former	60 542	10.2
Current	212 295	35.9
Missing	25 629	4.3

**Smoking during pregnancy**		
No	504 128	85.3
Smoker	61 021	10.3
Missing	25 629	4.3

**IMD quintile based on GP location**		
1 (least deprived)	104 166	17.6
2	94 699	16.0
3	110 142	18.6
4	124 141	21.0
5 (most deprived)	157 630	26.7

**Region**		
North East	11 230	1.9
North West	71 945	12.2
Yorkshire & The Humber	20 908	3.5
East Midlands	23 513	4.0
West Midlands	54 894	9.3
East of England	54 143	9.2
South West	52 368	8.9
South Central	67 521	11.4
London	56 796	9.6
South East Coast	56 388	9.5
Northern Ireland	21 181	3.6
Scotland	49 389	8.4
Wales	50 502	8.5

a *Other: eating and personality disorders. CMI = common mental illness. IMD = Index of Multiple Deprivation. SMI = serious mental illness.*

### Child atopy and maternal mental illness

#### Asthma

Overall, the incidence of asthma diagnosis was highest among children exposed to other maternal mental illness (18.37 per 1000 person–years) (Table 2); both other maternal mental illness (crude HR 1.39, 95% CI = 1.27 to 1.51) and CMI (crude HR 1.31, 95% CI = 1.29 to 1.34) increased the risk of childhood asthma. After adjusting for potential confounders (aHR 1.19, 95% CI = 1.16 to 1.22, model 1) and adjustment for smoking (aHR 1.17, 95% CI = 1.15 to 1.20, model 2), only CMI was associated with an increased risk of asthma.

**Table 2.** Unadjusted and adjusted HRs showing the association between atopy and maternal mental illness (*N* = 590 778 children)

**Condition**	**Person–years, 1000**	**Cases, *n***	**Rate (95% CI) per 1000 person–years**	** Unadjusted HR**	** *P*-value **	**Adjusted HR model 1^a^ (95% CI) **	** *P*-value **	**Adjusted HR model 2^b^ (95% CI) **	***P*-value**
**Asthma**									
Unexposed	2352.68	30 060	12.78 (12.63 to 12.92)	Ref	—	Ref	—	Ref	—
CMI	1213.67	19 394	15.98 (15.76 to 16.21)	1.31 (1.29 to 1.34)	<0.0001	1.19 (1.16 to 1.22)	<0.0001	1.17 (1.15 to 1.20)	<0.0001
SMI	18.30	263	14.37 (12.74 to 16.22)	1.11 (0.98 to 1.25)	0.107	1.04 (0.90 to 1.21)	0.566	1.04 (0.90 to 1.20)	0.629
Addiction	34.65	498	14.37 (13.15 to 15.69)	1.12 (1.02 to 1.22)	0.015	1.01 (0.91 to 1.12)	0.883	0.98 (0.88 to 1.09)	0.713
Other^c^	29.24	537	18.37 (16.88 to 19.99)	1.39 (1.27 to 1.51)	<0.0001	1.08 (0.97 to 1.20)	0.163	1.08 (0.97 to 1.20)	0.176

**Allergic rhinitis**									
Unexposed	2429.48	19 070	7.85 (7.73 to 7.96)	Ref	—	Ref	—	Ref	—
CMI	1288.28	13 659	10.60 (10.43 to 10.78)	1.22 (1.19 to 1.25)	<0.001	1.16 (1.13 to 1.20)	<0.001	1.17 (1.13 to 1.21)	<0.001
SMI	19.64	192	9.78 (8.49 to 11.26)	1.02 (0.88 to 1.18)	0.766	0.96 (0.80 to 1.14)	0.608	0.97 (0.81 to 1.15)	0.714
Addiction	37.10	338	9.11 (8.19 to 10.13)	0.95 (0.85 to 1.07)	0.404	0.91 (0.80 to 1.05)	0.188	0.92 (0.80 to 1.05)	0.222
Other^c^	31.67	356	11.24 (10.13 to 12.47)	1.19 (1.07 to 1.33)	0.001	1.04 (0.91 to 1.19)	0.570	1.05 (0.92 to 1.20)	0.490

**Eczema**									
Unexposed	1803.80	130 512	72.35 (71.96 to 72.75)	Ref	—	Ref	—	Ref	—
CMI	922.01	49 096	53.25 (52.78 to 53.72)	1.01 (1.00 to 1.02)	0.091	1.00 (0.99 to 1.01)	0.985	1.02 (1.00 to 1.03)	0.023
SMI	14.64	642	43.86 (40.60 to 47.39)	0.90 (0.84 to 0.98)	0.010	0.91 (0.83 to 1.00)	0.040	0.93 (0.85 to 1.01)	0.093
Addiction	28.04	1125	40.12 (37.84 to 42.53)	0.82 (0.78 to 0.87)	<0.001	0.87 (0.81 to 0.93)	<0.0001	0.91 (0.85 to 0.97)	0.007
Other^c^	22.32	1240	55.56 (52.55 to 58.74)	1.06 (1.00 to 1.12)	0.046	1.06 (0.99 to 1.13)	0.101	1.06 (0.99 to 1.14)	0.083

**Food allergies**									
Unexposed	2493.37	6864	2.75 (2.69 to 2.82)	Ref	—	Ref	—	Ref	—
CMI	1359.41	2809	2.07 (1.99 to 2.14)	0.95 (0.90 to 0.99)	0.019	0.96 (0.91 to 1.01)	0.133	0.99 (0.94 to 1.05)	0.854
SMI	20.73	46	2.22 (1.67 to 2.96)	1.10 (0.83 to 1.47)	0.501	1.04 (0.75 to 1.46)	0.796	1.08 (0.78 to 1.51)	0.641
Addiction	39.27	40	1.02 (0.75 to 1.39)	0.51 (0.37 to 0.69)	<0.001	0.59 (0.41 to 0.84)	0.004	0.65 (0.45 to 0.93)	0.018
Other^c^	33.85	70	2.07 (1.64 to 2.61)	0.99 (0.78 to 1.25)	0.924	1.08 (0.82 to 1.43)	0.569	1.11 (0.84 to 1.47)	0.465

a *Adjusted for maternal history of atopic disease, antibiotic use during pregnancy, maternal age, child sex, child ethnicity, birth season, birth year practice IMD, and practice region.*

b *Adjusted for all variables in model 1, plus maternal smoking.*

c *Other: eating and personality disorders. CMI = common mental illness. HR = hazard ratio. IMD = Index of Multiple Deprivation. Ref = reference. SMI = serious mental illness.*

#### Allergic rhinitis

Incidence was highest in children exposed to other maternal mental illness (11.24 per 1000 person–years) (Table 2). Both other maternal mental illness (aHR 1.04, 95% CI = 0.91 to 1.19, model 1) and CMI (aHR 1.16, 95% CI = 1.13 to 1.20, model 1) increased the risk of allergic rhinitis. After adjustment for smoking only, maternal CMI increased the risk of allergic rhinitis (aHR 1.17, 95% CI = 1.13 to 1.21, model 2). Children exposed to maternal CMI had a similar risk of asthma and allergic rhinitis.

#### Eczema

Unexposed children had the highest incidence of eczema (72.35 per 1000 person–years) (Table 2). Children exposed to SMI had a 9% reduced risk of developing eczema compared with unexposed children (aHR 0.91, 95% CI = 0.83 to 1.00). Children exposed to maternal addiction also had reduced risk of eczema (aHR 0.87, 95% CI = 0.81 to 0.93, model 1).

The reduced risk of eczema remained for children exposed to SMI (aHR 0.93, 95% CI = 0.85 to 1.01) and maternal addiction (aHR 0.91, 95% CI = 0.85 to 0.97), after adjustment for confounders and maternal smoking (model 2) (Table 2).

#### Food allergies

Unexposed children had the highest incidence of food allergies (2.75 per 1000 person–years) (Table 2). Compared with unexposed children, children exposed to CMI had a 5% reduced risk (crude HR 0.95, 95% CI = 0.90 to 0.99), which was inconclusive after adjusting for potential confounders. Children exposed to maternal addiction had a reduced risk of allergy (crude HR 0.51, 95% CI = 0.37 to 0.69) that persisted following adjustment for confounders (aHR 0.65, 95% CI = 0.45 to 0.93, model 2).

#### Covariates.

Overall, male children, children in the most deprived quintile, and those exposed to maternal asthma, maternal antibiotic use during pregnancy, or maternal smoking had increased risk of asthma. In contrast, developing eczema and food allergies was associated with children being less deprived, without exposure to maternal smoking (see Supplementary Tables S1 and S2).

### Hospital admission for asthma and food allergy

There was a 29% increased risk of admission to hospital for asthma among children exposed to CMI (aHR 1.29, 95% CI = 1.20 to 1.38, model 1, Table 3). For the other types of maternal mental illness there was weaker evidence of an association after adjusting for potential confounders (for example, maternal addiction) (aHR 1.27, 95% CI = 0.95 to 1.71, *P* = 0.113, model 1).

**Table 3.** Unadjusted and adjusted HRs showing the association between maternal mental illness and offspring admission to hospital for asthma (*N* = 359 611 children)^a^

**Condition**	**Person–years, 1000**	**Asthma cases, *n* **	**Asthma rate (95% CI) per 1000 person–years **	**Unadjusted HR (95% CI) **	** *P*-value **	**Adjusted HR model 1^b^ (95% CI) **	***P*-value **	**Adjusted HR model 2^c^ (95% CI) **	***P*-value**
**Unexposed**	1538.59	2059	1.34 (1.28 to 1.40)	Ref	—	Ref	—	Ref	—
**CMI**	797.58	1412	1.77 (1.68 to 1.87)	1.46 (1.37 to 1.57)	<0.0001	1.29 (1.20 to 1.38)	<0.0001	1.26 (1.17 to 1.36)	<0.0001
**SMI**	11.70	15	1.28 (0.77 to 2.13)	0.98 (0.59 to 1.64)	0.951	0.76 (0.45 to 1.27)	0.296	0.77 (0.46 to 1.28)	0.315
**Addiction**	21.41	46	2.15 (1.61 to 2.87)	1.72 (1.29 to 2.30)	<0.0001	1.27 (0.95 to 1.71)	0.113	1.20 (0.89 to 1.62)	0.236
**Other^d^**	19.88	45	2.63 (1.69 to 3.03)	1.68 (1.25 to 2.25)	0.001	1.28 (0.95 to 1.72)	0.103	1.29 (0.96 to 1.74)	0.088

a *Owing to CPRD’s small cell policy, patient counts with fewer than five observations have been suppressed. Therefore, food allergy-related admission to hospital results are not shown in this Table.*

b *Adjusted for maternal history of atopic disease, antibiotic use during pregnancy, maternal age, child sex, child ethnicity, birth season, birth year practice IMD, and practice region.*

c *Adjusted for all variables in model 1, plus maternal smoking.*

d *Other: eating and personality disorders. CMI = common mental illness. CPRD = Clinical Practice Research Datalink. HR = hazard ratio. IMD = Index of Multiple Deprivation. Ref = reference. SMI = serious mental illness.*

The results were inconclusive for admissions to hospital related to food allergy (data not shown due to a small cell count).

### Sensitivity analysis

Excluding children with cases of infantile eczema did not change the risk of childhood eczema associated with CMI (aHR 1.06, 95% CI = 1.02 to 1.10, model 2). However, results for SMI, addiction, and other maternal mental illness were less conclusive (see Supplementary Table S3). Given the inverse association between maternal addiction and eczema, post hoc*,* the authors examined whether eczema was more severe in children exposed to maternal addiction; and found weak evidence of an increased risk of admission to hospital for skin disorders among children with eczema exposed to maternal addiction (3.57 versus 2.81, admission rate per 1000 person–years, aHR = 1.47 [model 2], 95% CI = 0.98 to 2.22, *P* = 0.063, Supplementary Table S4).

## DISCUSSION

### Summary

To the authors’ knowledge, this is the first UK population-based study to examine the association between maternal mental illness and risk of the range of atopic disorders in children. The main findings were that children exposed to maternal CMI were at the highest risk of developing asthma and allergic rhinitis. Childhood exposure to maternal addiction or SMI reduced the risk of childhood eczema and exposure to maternal addiction reduced the risk of food allergy. Also, counter to the authors’ hypothesis, children of mothers with SMI showed no excess risk of atopic disorders.

Atopic disease was more common in males, children of ethnic Asian heritage, and those exposed to antibiotics during pregnancy or maternal atopy (see Supplementary Table S1).

As anticipated, maternal smoking was associated with childhood asthma but, unexpectedly, with reduced risk for eczema and food allergies.

The influence of deprivation varied by outcome: children from the most deprived households had the highest risk of asthma and lowest risk of eczema. This pattern was apparent for food allergies, but less so for allergic rhinitis. Finally, asthma- related admission to hospital was greater among children exposed to maternal CMI compared with unexposed children.

### Strengths and limitations

The CPRD provides a large enough cohort to estimate, with precision, links between individual maternal mental illnesses and offspring atopic disorders. However, some limitations remain. It was not possible to capture eczema severity, which is potentially vital to explain why SMIs are associated with reduced childhood eczema in primary care. The effects of maternal mental illness on risk of atopy reported here do not account for unmeasured confounders such as housing quality and paternal mental health/atopy. Important risk factors other than smoking that might be on the causal pathway, such as air pollution, were not recorded.

The study could only measure geographical region and area-level indices of deprivation. Effects of mental illness in ethnicities other than White British mothers may be underestimated because they are relatively underrepresented in this primary care cohort.^29^ Finally, using HES linkage to examine risk of admission to hospital excluded many mother–child pairs in England and children from Scotland, Wales, or Northern Ireland, since HES linkage is only available for NHS England and some English general practices do not consent to this linkage.

### Comparison with existing literature

An increased risk of asthma and allergic rhinitis in children of mothers with CMI replicates other large cohort studies.^18^^,^^19^^,^^30^ The current study has extended these findings by demonstrating the specificity of this association, suggesting shared factors increase maternal depression/anxiety and childhood atopy,^31^ which may be expressed in utero.^12^

The current study does not replicate smaller studies reporting greater risk of eczema in children exposed to maternal CMI.^15^^,^^17^^,^^32^ However, removing those with infantile eczema unmasked excess eczema in children exposed to maternal CMI; and maternal smoking and socioeconomic deprivation explained some of the association — children from the most deprived areas manifest reduced risk of eczema/food allergies. Eczema is common in infancy (0–3 years) and likely to be mild and self- limiting. It could be that mothers in higher socioeconomic groups are more health literate and spot eczema earlier than women who live in more deprived circumstances.^33^

As a group, atopic disorders share an immune aetiology;^34^ however, the current results suggest heterogeneity in the pathways for different disorders. Similar heterogeneity is observed for parental mental illness and autoimmune disease.^35^

It is increasingly recognised that infantile and pregnancy exposure to diversity in the microbiota is important for development of immune responses^36^ and that diet is key to maintaining those through life.^37^ This might explain why children exposed in utero to antibiotics develop more atopy. Also, if household crowding is more common in those with serious mental illness or addiction this is one way early immune responses could be strengthened and may explain why children in these settings develop less atopy.^12^

Differences in maternal health anxiety may also clarify some of the different risk associations, for example, mothers with anxiety disorders being more vigilant and/or reporting more symptoms. In the current study, the authors report that children exposed to maternal addiction are less likely to be seen in primary care for eczema, and cautiously report an increased likelihood of admission to hospital for eczema. Milder disorders may be undetected by ill mothers; mothers living in deprived areas, ethnic minority, and migrant mothers may be less able to attend/arrange a GP appointment because of limited knowledge, lack of English, access to transport, or childcare.^29^^,^^38^

### Implications for research and practice

Public health policies and practice guidelines, including the Healthy Child Programme,^39^ should be modified to tailor support to mothers with mental illness whose health literacy may be compromised. For instance, children were at high risk of developing asthma following exposure to maternal CMI; but also if they were exposed to maternal smoking during their lifetime or to antibiotics in utero; and if they lived in the most deprived areas. Therefore, expanding preventive health programmes to target vulnerable women ante- and postnatally (for example, smoking cessation), and tailoring information specifically to their needs stands out as a clear implication of the authors’ findings for general practice.

Such tailored information is important because, increasingly, the evidence suggests that people with mental illness underestimate their risk of poor physical health, and are less likely to adopt preventive health measures or be able to change behaviour following widespread public health campaigns.^40^ This means that we run the risk of increasing health inequalities if those at greatest risk are unable to access the benefits of available public health care. Training for healthcare providers in general practice who socially prescribe and signpost (for example, making every contact count),^41^ might help reduce the consistently high rates of smoking in mothers with mental illness.

In conclusion, common mental illnesses in mothers increases asthma and allergic rhinitis in offspring. Aetiological mechanisms in atopic disorders remain elusive, but this and other findings make it likely that maternal mental illness plays a significant aetiological role. Future research should triangulate findings from studies of different designs to discern causality and mitigate flaws inherent in each, to better account for unmeasured confounding.^42^

Evidence of the population effects of providing tailored support to mothers with mental illness is needed. Evidence of the impact of implementing tailored support for mothers on child health outcomes is lacking, and data are limited to a few randomised controlled trials with mixed results, primarily in low and middle-income countries. Such tailoring would allocate resources more equitably and might deliver significant health and economic benefits for all.

## Data

The clinical codes used in this study are published on github.^24^ Access to data are available only once approval has been obtained through the individual constituent entities controlling access to the data. The primary care data can be requested via application to the CPRD (www.cprd.com).
